# Error-Correcting Output Codes in Classification of Human Induced Pluripotent Stem Cell Colony Images

**DOI:** 10.1155/2016/3025057

**Published:** 2016-10-26

**Authors:** Henry Joutsijoki, Markus Haponen, Jyrki Rasku, Katriina Aalto-Setälä, Martti Juhola

**Affiliations:** ^1^School of Information Sciences, University of Tampere, Kanslerinrinne 1, 33014 Tampere, Finland; ^2^BioMediTech, University of Tampere, Biokatu 12, 33520 Tampere, Finland; ^3^School of Medicine, University of Tampere, Biokatu 12, 33520 Tampere, Finland

## Abstract

The purpose of this paper is to examine how well the human induced pluripotent stem cell (hiPSC) colony images can be classified using error-correcting output codes (ECOC). Our image dataset includes hiPSC colony images from three classes (bad, semigood, and good) which makes our classification task a multiclass problem. ECOC is a general framework to model multiclass classification problems. We focus on four different coding designs of ECOC and apply to each one of them *k*-Nearest Neighbor (*k*-NN) searching, naïve Bayes, classification tree, and discriminant analysis variants classifiers. We use Scaled Invariant Feature Transformation (SIFT) based features in classification. The best accuracy (62.4%) is obtained with ternary complete ECOC coding design and *k*-NN classifier (standardized Euclidean distance measure and inverse weighting). The best result is comparable with our earlier research. The quality identification of hiPSC colony images is an essential problem to be solved before hiPSCs can be used in practice in large-scale. ECOC methods examined are promising techniques for solving this challenging problem.

## 1. Introduction

Human induced pluripotent stem cells (hiPSCs) have gained a lot of attention during the last decade. All began in 2006 when Takahashi and Yamanaka [1] reported in an article published in Cell journal that mouse embryonic and adult fibroblasts can be reprogrammed back to stem cells. These stem cells were called induced pluripotent stem cells (iPSCs). Overall, this result was a groundbreaking innovation which reformed the regenerative medicine totally and pointed to a new direction for the modern medicine. By this means patient specific drug therapy, disease modeling, and tissue repairing, for example, can be commonplace procedures in near future since iPSCs are “capable of differentiating all germ layers (endoderm, mesoderm and ectoderm) and the germline but not extra embryonic tissues” as Amabile and Meissner stated in [2].

The actual reprogramming process of somatic cells was performed by introducing four genes encoding transcription factors called Oct3/4, Sox2, Klf4, and C-Myc [1, 3]. The progress of iPSC research did not end by the findings of the year 2006 article but in the following year Takahashi et al. [3] reproduced the reprogramming process for human fibroblasts and the stem cells were called human induced pluripotent stem cells. The invention of the reprogramming process itself was a very important issue but it also alleviated the strong ethical debate that the use of stem cells in medical treatments had confronted before. Now we were able to use a patient's own cells in medical treatments.

Although the necessity of hiPSCs is evident from the medical perspective, the demand of computer science with hiPSCs might not be clear at first sight. Currently, the use of hiPSCs in medicine can be considered still to be at the theoretical level and there are several challenges which must be solved before they are used in large-scale in practice. Some of the problems are biomedical (see, e.g., [4]) but discussion related to biomedical problems is out of the scope of this paper. Instead, we focus on problems which can be solved computationally and are encountered in practice among practitioners and researchers in biomedicine who examine and deal with hiPSCs in their daily routines. The nature of these problems is usually practical but the solution may require highly sophisticated methods from the fields of machine learning, image analysis, or signal processing, for instance.

Quality identification of hiPSC colonies (colony is a group of individual hiPSCs) is one of the daily tasks that personnel working in biomedicine perform. The workflow of quality identification that is done in practice can be described in two stages:

- Recognize the colony using the microscope.
- Determine the quality of the colony.

Currently, quality identification of hiPSCs is performed manually which is impossible to carry out in large-scale and causes great workload for the personnel of a laboratory. However, quality identification is an essential process to do because abnormal hiPSC colonies cannot be used for any purposes such as disease modeling and patient specific drug therapy. Thus, we have a clear motivation to automate the quality identification process. Automation will ease the workload of the personnel and offers an objective tool for quality identification.

When considering the workflow more specifically, we see that it has a classical pattern recognition structure where the first part is the recognition phase and the other part considers classification. The recognition part returns to image analysis and isolating the colony area from the image after the colony has been imaged using a camera. Currently, the recognition of colonies is done manually by human experts. Determining the quality of the hiPSC colony instead can be performed using supervised machine learning methods. We restrict our focus on the machine learning stage and the image analysis part will be left outside of this paper. Although the automation of quality identification of hiPSC colonies will be necessary action in the near future, the need of human expertise does not disappear. In order to use supervised machine learning techniques we need properly constructed image set which is labeled by the human expert. In the construction of such set the knowledge of human experts will be required.

The specific goal of our study is to examine how well hiPSC colony images can be classified using error-correcting output codes (ECOC). More specifically, we want to investigate whether or not the use of ECOC can improve the accuracy compared to our earlier study [5] on the same dataset. Overall, ECOC [6–8] is a general framework to model multiclass classification problems by dividing it to a set of binary classification problems.

Since ECOC is not attached to any specific classification method, it can be used with various classification methods. For this paper we restricted our focus on the following classification methods to be used with ECOC:

(i) *k*-Nearest Neighbor (*k*-NN) searching method
(ii) Discriminant analysis based solutions
(iii) Naïve Bayes
(iv) Classification tree

From the selected classification methods particularly *k*-NN is interesting because it obtained good results in [5]. The aforementioned classification methods are themselves multiclass classifiers and tested in [5]. However, now we examine whether or not we can improve our results using selected classifiers in the context of ECOC. In ECOC there are numerous ways (in other words, coding designs) to reduce a multiclass classification problem into a set of binary classification problems. From the possible coding design alternatives we chose four approaches to be used:

- One-vs-all (OVA)
- One-vs-one (OVO)
- Ordinal (ORD)
- Ternary complete (TER)

The motivation to apply ECOC framework in the context of the hiPSC colony image classification can be explained by its good performances in many applications such as cloud classification [9], text classification [10], face verification [11, 12], face detection [13], and ECG beats classification [14] and in mapping criminal phenomena [15].

Classification of hiPSC colony images has recently been studied in [5, 16–18]. The difference between the given references and our paper is significant. Firstly, Masuda et al. [18] had a binary classification problem between good and bad iPSC colonies whereas our problem is a multiclass problem between good and semigood and bad colony classes. Secondly, we use different Scaled Invariant Feature Transformation (SIFT) [19] based features in classification compared to [18] and [16, 17] in which only intensity histograms were used as features. Thirdly, although in [5] we used the same SIFT-based features and image dataset as in this study, in none of the earlier studies has ECOC framework been studied before and, hence, our research fulfills the missing gap. Fourthly, we have a significantly larger image dataset compared to [16–18].

The paper is organized as follows.  Section 2 describes briefly the ECOC framework and the classification methods used. In Section 3 a detailed description of experiments is given. The section consists of information about the dataset, data acquisition, feature extraction, classification procedure, and performance measures selected.  Section 4 presents the results and, finally, Section 5 is for discussion and Section 6 is for conclusions.

## 2. Methods

### 2.1. Classification Methods

We chose four classification methods to be used in this paper. These are *k*-NN method [20–22], linear and quadratic discriminant analysis variants [21–23], naïve Bayes [24, 25], and classification tree (more specifically CART algorithm) [22, 26]. There are issues which must be taken into account when the methods are used in practice. With *k*-NN the three main parameters are to be considered: the value of *k*, distance measure, and distance weighting function. We selected seven distance measures (Chebyshev, cityblock, correlation, cosine, Euclidean, standardized Euclidean, and Spearman distance measures) which were also successfully tested in [5, 16]. For distance weighting functions we chose equal, inverse, and squared inverse weighting [5, 16] and *k* values from 1 to 23 were tested. Only odd values were tested to prevent the possibility of a tie in the case of individual binary classifier.

Discriminant analysis based classification methods require the computing of covariance matrix. In linear discriminant analysis (LDA) we assume that all covariance matrices are equal [22, 23] whereas in quadratic discriminant analysis (QDA) the covariance matrix is evaluated for each class separately and they may differ from each other. There are different ways to use covariance matrix together with LDA and QDA in classification. We can use only diagonal entries of the estimated covariance matrix and the approach returns to naïve Bayes classification and can be called “diagLinear” or “diagQuadratic” [27]. A commonly encountered problem with the LDA and QDA based classification methods is the singularity of covariance matrix. In order to solve the problem of singularity we can use pseudoinverses. By this means obtained classifier is called “pseudoLinear” or “pseudoQuadratic” [27].

Naïve Bayes (NB) classifier is a well-known classification method. The basic formulation of NB classifier is simple and in many applications it works well. However, we can extend the use of NB classifier by using kernel smoothing density estimation [21, 27]. We applied altogether four different kernels (Gaussian, box, Epanechnikov, and triangle) in kernel smoothing density estimation.

### 2.2. Error-Correcting Output Codes

Error-correcting output codes are general classification framework which includes two stages: encoding and decoding [28–30]. Encoding phase consists of designing coding matrix CM where columns represent binary classifiers and rows indicate codewords for classes. Designing of a coding matrix can be made using binary coding and ternary coding [28]. In binary coding the elements of a coding matrix are CM_*ij*_ ∈ {−1,1} or CM_*ij*_ ∈ {0,1}, respectively, *i* = 1,2,…, *M* and *j* = 1,2,…, *N*_*c*_, where *M* is the number of classes and *N*_*c*_ is the number of binary classifiers. In the columns the division of values of −1 and 1 or 0 and 1, respectively, shows which classes are grouped together in each classifier. In ternary coding [6, 28], the elements of the coding matrix belong to the set {−1,0, 1}. In this approach values −1 and 1 have the same purpose as in binary coding but now 0 means that this specific class is excluded from the training of an individual binary classifier.

There are numerous strategies of how to construct the coding matrix. We chose four of the possible alternatives. The first approach is one-vs-all (OVA) [28] which uses binary coding and each one of the binary classifiers separates one class from the rest. Hence, the number of binary classifiers is only *𝒪*(*M*) where *M* is the number of classes. OVA is a very simple approach but from the computational perspective it is inefficient since the whole training set is needed in the case of each binary classifier. The coding matrix for OVA in the three-class classification problem can be found from Table 1.

The second coding design is one-vs-one (OVO) [29] which is another commonly used multiclass modeling method (Table 2). OVO coding design has several differences compared to OVA. Firstly, OVO applies ternary coding. Secondly, the individual binary classifiers are trained only with training data from classes *i* and *j* (*i* < *j* and *i*, *j* ∈ {1,2,…, *M*}). Thirdly, the total number of binary classifiers is *𝒪*(*M* ^2^). The coding matrix for OVO used in this paper can be seen from Table 2.

The third coding design is ordinal (ORD) [27] which has similarities with OVA. The number of binary classifiers is *M* − 1, that is, *𝒪*(*M*). Furthermore, every binary classifier uses the whole training data. In ORD approach the first binary classifier is trained to separate the first class from the rest whereas the second classifier separates the first two classes from the rest and so on until all *M* − 1 binary classifiers have been trained.  Table 3 presents the coding matrix for ORD in the three-class classification task.

The fourth coding design is called ternary complete (TER) [27] and it uses ternary coding.  Table 4 shows the coding matrix in the three-class classification problem and the total number of classifiers in TER approach is (3^*M*^ − 2^(*M*+1)^ + 1)/2 ∈ *𝒪*(3^*M*^). Compared to the aforementioned coding designs in the ternary complete design the number of classifiers is significantly higher and the training phase is more time-consuming in practice.

Besides OVA, OVO, ORD, and TER coding designs, there are also other choices which have been suggested in literature. Binary complete [14, 27] approach is analogous to TER approach but uses only binary coding. In binary complete coding design we construct all possible binary combinations without excluding any class. Hence, the number of individual binary learners is 2^(*M*−1)^ − 1 ∈ *𝒪*(2^*M*^) [14, 27]. However, the binary complete coding design is the same as OVA when the number of classes is three. Other known methods for designing a coding matrix are sparse random method [6, 29], dense random strategy [6], or discriminant error-correcting output codes [30]. For instance, sparse random and dense random strategies are more convenient for classification problems where the number of classes is high.

After the coding matrix has been built and individual classifiers have been trained, the next phase is to classify test examples. Each test example will be given for all classifiers which will predict an output for the example. When all outputs have been collected together, we have constructed a codeword for the test example. Then, we need to find out which one of the classes' codewords is the closest one to the test example's codeword. This stage is called decoding. There are numerous techniques for decoding and Escalera et al. [28, 31] presented an extensive review on this issue. The most common ways for decoding are to use Hamming decoding [31], inverse Hamming decoding [32], and Euclidean decoding [30] according to Escalera et al. [28]. In this paper we used Hamming loss function for binary learners and loss-weighted decoding [31] for aggregating the individual losses. Detailed formulas for Hamming and loss-weighted decodings can be found from [28, 31].

## 3. Experiments

### 3.1. Dataset Description

**Figure 1:**
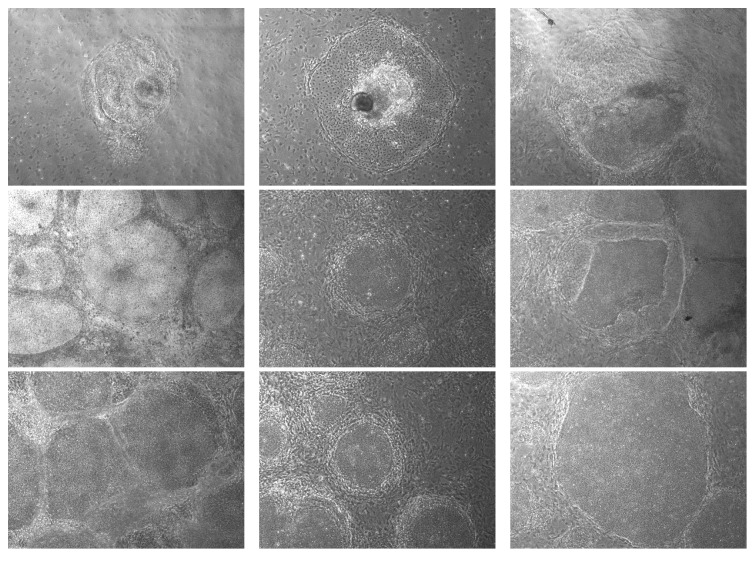
Example images on iPSC colonies from classes bad, semigood, and good. Images on the first row are from the class bad, the second row images are from the class semigood, and the third row indicates colonies from the class good. Images are scaled to have width and height of 1.5 in.

Our dataset includes altogether 173 hiPSC colony images from three classes: bad, semigood, and good. From these classes semigood class can be considered as a transition phase from good to bad. More specifically, we have 41 images belonging to class bad, 74 images in class good, and the rest of the images (58) in semigood class. Images cannot be connected to any specific patient. Three example images from each class can be seen from Figure 1.

The study was approved by the ethical committee of Pirkanmaa Hospital District (R08070). iPSC lines were established using the same approach as given in [1] and cell lines were characterized for their karyotypes and pluripotency as described in [33]. Categorization of the iPSC colony images was performed as follows [5, 16, 17]:

- Good colonies have rounded shape, translucent even color, and defined edges.
- Semigood colonies have clear edges but include changes in color and structure.
- Bad colonies have partially lost edge structure, vacuole could sometimes be seen, and areas of three-dimensional structures were observed.

### 3.2. Data Acquisition

Image data acquisition followed the guidelines given in [5, 16, 17]. We used hiPSCs and colonies were photographed between 5 and 7 d of their weekly growth cycle since within this time frame better visualization of the colonies can be obtained. The following setup was used in observation and image acquisition:

(i) Nikon Eclipse TS100 inverted routine microscope with an attached heating plate
(ii) Imperx IGV-B1620 M-KC000 camera which was attached to the microscope and connected to a laptop
(iii) Laptop that included JAI Camera Control Tool software
(iv) Images with resolution of 1608 × 1208 (width × height)

Imaging process may include problems related to lighting and sharpness of an image, for instance. These problems, however, were minimized since only one expert performed the actual imaging process. Hence, the variability of lighting and sharpness of an image was minimized and the settings were fixed to during imaging sessions. Since the images were taken during several sessions, it may have caused small differences in the images. The position of growing hiPSC colonies was mostly in the center of the image but sometimes a growing colony was near the edge of the well which produced some distortion in the lighting. Finally, the imaged hiPSC colonies were classified to one of the three aforementioned classes.

### 3.3. Feature Extraction

In this study we used Scaled Invariant Feature Transformation (SIFT) [19] based features. We applied basic SIFT algorithm and not the dense SIFT [34, 35] variation. SIFT algorithm is a well-known algorithm and a detailed presentation about it can be found from [19]. We performed the extraction of SIFT descriptors and frames using VLFeat 0.9.18 with default settings [36]. SIFT feature extraction from an image produces descriptors and their corresponding frames. Descriptors are presented as vectors of 128 dimensions. We used in feature extraction a technique presented in [5]. This technique has two phases and is done for every image separately. The first stage is to extract SIFT descriptors from an image. The other stage is to evaluate a mean descriptor from the descriptors gained in the first stage. Thus, every image is represented by a mean SIFT descriptor and it can be used in classification. This “mean SIFT descriptor” approach improved classification results in [5] compared to [16, 17] and this inspired us to use the same approach again in this paper. When mean SIFT descriptors were obtained, the dataset to be used in classification was size (excluding column of class labels) of 173 × 128 (rows × columns). Before the actual classification we performed simple preprocessing by standardizing the columns of dataset to have a mean of zero and unit variance. After standardization we scaled the columns (features) to [−1,1] interval.

### 3.4. Classification Procedure

Classification was performed using either leave-one-out (LOO) or nested leave-one-out technique (NLOO). LOO was used when there were not any parameters to be estimated in terms of classification method. This was the case when discriminant-based classification methods, classification tree, and naïve Bayes classifier were used with different ECOC coding designs.

In the case of *k*-Nearest Neighbor method there are several parameters and these were explained in Section 2.1. In addition, in Section 2.1 parameter values tested were presented. From the parameters *k* value must be estimated and this was performed using NLOO technique. NLOO includes two for loops where the outer loop is for model evaluation and the inner loop is for model selection. The following classification procedure is repeated with respect to all examples in the dataset:

(1) In outer loop exclude one example from the dataset and form a test set on this example. This example is left for model evaluation. The rest of the examples form training set.
(2) Perform LOO procedure for the training set and repeat it with each *k* value.
(3) Evaluate the accuracy of the training set for each *k* value tested. Select the *k* value which gained the highest accuracy.
(4) Train *k*-NN binary classifiers by utilizing the whole training set and optimal *k* value and predict the class label for the test example (obtained in step (1)).

A consequence of NLOO is that for each training set the optimal *k* value may vary. In addition, from the computational perspective, NLOO is the most time-consuming classification procedure. However, with NLOO we can maximize the size of the training set which is an asset when dealing with rather small datasets as ours. The hardware used in classification was a desktop computer having Intel i7-3960X 3.5 GHz processor and 32 GB RAM. All the experiments were made using Matlab 2015b and Statistics and Machine Learning Toolbox, Image Processing Toolbox, and Parallel Computing Toolbox with Win7 operating system.

### 3.5. Performance Measures

We have chosen three performance measures which are true positive rate (TPR) (also known as sensitivity), true positive (TP), and accuracy (ACC). True positive rate describes the proportion of correctly classified examples from a specific class. True positive presents the number of correctly classified examples from a specific class. Accuracy describes the proportion of correctly classified examples from all classes together. From these performance measures accuracy and true positive rate can be given also in percentages. Formulas for the performance measures can be found, for example, from [23]. Other performance measures were not chosen since majority of the performance measures are designed for binary classification problems (e.g., ROC, AUC, and *F*_1_-score) and for our purposes the aforementioned three performance measures are adequate.

## 4. Results

Table 5 shows the results of discriminant analysis variants and classification tree. Overall, it can be seen that the results are largely dispersed. Accuracies spread from less than 25% to over 57%. Only with diagLinear and classification tree above 50% accuracy was obtained. The best choice according to Table 5 was classification tree with OVO coding design having 57.2% accuracy and outperforming results in [16, 17]. Also, with the ternary complete coding design classification tree obtained above 50% accuracy. If we examine more closely classification tree results, we notice that none of the classes was classified with very high TPRs. The best one was class “good” where close to 68% for TPR was obtained. In classes “bad” and “semigood” TPRs were mostly in balance between each other. We must also remember that class “good” is the most frequent in the dataset which may have influenced the results. However, most of the TPRs were left below 50% which indicates how small differences are between classes and the misclassification may happen very easily.

The rest of the classification methods in Table 5 could not compete with classification tree results. Linear discriminant analysis (LDA) with all coding designs stayed below 40% in accuracies and most of the TPRs. This is a poor result and indicates that the classes are not easily separable. For diagLinear instead the results were improved radically partially. A general insight to the results shows that accuracy and TP(R)s for class “good” gained better results compared to LDA results whereas for classes “bad” and “semigood” the results stayed pretty much at the same level as in LDA case. A noticeable detail is that with OVA coding design diagLinear obtained the highest TP(R) combination (59 (79.7%)) for class “good.” A runner-up accuracy (52.0%) of Table 5 was also obtained with diagLinear.

The last discriminant-based classification method was called “pseudoQuadratic.” The results of pseudoQuadratic were dichotomous in terms of coding designs. For OVA and ORD coding designs class bad was classified perfectly but other classes were misclassified totally. In the case of OVO and TER coding designs accuracies were around 41.0% and none of the classes were misclassified totally. Furthermore, the highest TP(R) (31 (53.4%)) was obtained for class “semigood” when TER coding design was used.

Table 6 presents the results of naïve Bayes (NB) variants when different ECOC coding designs were used. If we consider results column-wise, we see that classes “bad” and “semigood” were the most difficult to recognize. In both classes the best TPRs were below 42.0%. More specifically, the highest TP(R)s for classes “bad” and “semigood” were 17 (41.5%) and 19 (32.8%). However, class “good” was well recognized since the topmost TP(R) was 65 (87.8%). Obtained TP(R)s are very good results but we need to keep in mind that NB is a classifier which relies heavily on probabilities and for class good a priori probability is the highest one among all classes. This certainly may have an effect on the results. For accuracies, 53.8% was achieved using OVA coding design and basic NB classifier with normal distribution assumption. We also tested four different kernels in kernel smoothing density estimation but these did not bring any improvement.

The last four tables (Tables 7 [Table tab8] [Table tab9]–10) concern the results of *k*-Nearest Neighbor searching (*k*-NN) method. We have separated the results of every coding design into its own table to ease the analysis.  Table 7 presents the interesting results of the OVA coding design with the *k*-NN variations. The highest accuracy (61.8%) is better than in previous result tables and when examining accuracies we notice that three methods obtained above 60.0% accuracy whereas in Tables 5 and 6 none of the methods gained above 60.0% accuracy. Class “good” is again the best identified class and TP(R)s of 62 (83.8%) were achieved. This is a very good result and indicates that class good can be separated well from the rest of the classes. In the case of class “bad” TP(R) combination 28 (68.3%) was gained and it was also an improvement compared to naïve Bayes results in Table 6 and majority of the results in Table 5. For class “semigood” situation was slightly different since the best TP(R) combination (27 (46.6%)) was not so good as with other classes. Nevertheless, the best result in this class was still better than the majority of the results of class “semigood” in Tables 5 and 6.

Table 8 depicts the results of *k*-NN classifiers with OVO coding design. There are several similarities in the results compared to Table 7. Firstly, class “good” was again the best recognized class within the dataset having 64 (86.5%) TP(R)s. Secondly, the best accuracy (60.7%) was only around 1% lower than in Table 7 and it was obtained with the same distance measure. Thirdly, class “semigood” was the most difficult class to classify and 29 (50.0%) TP(R)s were gained at maximum. For class “bad” a significant drop happened in TP(R)s. Now, the best TP(R) combination was 24 (58.5%) and it was obtained by three distance measures (cityblock, Euclidean measure, and standardized Euclidean). The actual difference in class “bad” compared to Table 7 results was around 10.0% which can be considered to be significant. The reason behind the decrease may lie in the OVO coding design itself where individual classifiers separate only two classes from each other whereas in OVA all classes are included in all classifiers.

Table 9 represents the results of *k*-NN classifier and ordinal (ORD) coding design. Now, the best accuracy was 61.8% similarly as in Table 7. Moreover, for class “good” 64 (86.5%) TP and TPRs were achieved and these were identical which was the topmost result in Table 8. However, now the best result was achieved by the same configuration as the highest accuracy. Cityblock distance measure together with equal and inverse weightings got the best TP and TPR (23 (56.1%)) which was in line with Table 8 results for class “bad.” Furthermore, for class “semigood” the same configuration as in Table 8 achieved the best TP(R) combination (26 (44.8%)). Hence, we can say that the results of ORD coding design were a mixture of OVA and OVO results.

The results of *k*-NN classifier with ternary complete coding design can be seen from Table 10. The same distance measures were the best alternatives as for OVA and OVO coding designs. Standardized Euclidean with equal and inverse weightings achieved 25 (61.0%) TP(R). In addition, standardized Euclidean was the best choice with respect to accuracies. Accuracy of 62.4% was the best accuracy when taking into account all accuracies in this paper. Moreover, it is an improvement over 7% to accuracies obtained in [16, 17] with a smaller hiPSC image dataset. When examining classes “good” and “semigood” more closely, we notice that class “good” was again the best recognized class having 61 (82.4%) TP(R). For class “semigood” the same level of result was gained as in Tables 7 [Table tab8]–9. This time the highest TP(R) was obtained by Euclidean measure and inverse weighting being 28 (48.3%). Overall, class “semigood” was difficult to classify and one reason behind it may be that “semigood” class includes greater variety between colonies. Some of the colonies might be closer to good colonies and some other colonies may be closer to bad colonies.

## 5. Discussion

Error-correcting output codes are a state-of-the-art technique when modeling multiclass classification problems. ECOC has been used in many applications and showed good performance. ECOC was developed originally to be used with binary coding but was extended to ternary coding which certainly has increased the interest towards ECOC among both practitioners and researchers.

The use of ECOC is a novel approach in the context of classification of hiPSC colony images. By thorough examination of different coding designs and classification methods we obtained promising results. The highest accuracy was 62.4% and it was a clear improvement compared to our earlier studies in [16, 17]. Moreover, accuracy (62.4%) gained is the same as we obtained in [5] using *k*-NN without ECOC. We also achieved improvement on classification tree and naïve Bayes results using ECOC framework compared to [5] where these classification methods were used without ECOC. This gives positive attitude towards ECOC and its application in hiPSC colony image classification. Despite the improvement more work has to be made before the accuracy is at such level that the methods developed can be used in practice in large-scale. Challenges with the hiPSC colony images are diverse and the choice of classification method is the final link in a long chain of processes.

When talking about hiPSC colony images everything begins in the image analysis related issues which is now done manually by human experts: how do we find the colony area from the image automatically or what kind of cleaning method would be the best one for removing the disturbing artefacts from the image. These tasks are problematic since the edges of colonies are not necessarily clearly seen and hiPSC colony images are sensitive to any image processing techniques. In addition, colonies are surrounded by feeder cells and sometimes these can penetrate the colony. These two things have an effect on feature extraction. For instance, we used SIFT feature extraction method which finds location, scale, and rotation invariant descriptors from the “keypoints” of images. Due to feeder cells some of the descriptors may be recognized from feeder cells whereas our interest is in the colonies. Since we described the image as a mean vector of SIFT descriptors, feeder cells and other possible artefacts may have influence on the mean vector and, thus, on classification results.

The results obtained showed that class “good” was the best recognized class in a dataset. A possible explanation for the separability of class “good” might be that good colonies differ morphologically from other classes' colonies. These facts may have influenced the feature extraction and, hence, classification. In Section 3.1 decision criteria for each colony class were given. For class “good” rounded shape and defined edges were evident whereas the changes in structure, color, and edges were seen on other classes. Throughout the results class “semigood” was the most difficult class to be classified. A rational reason for the observation can be that class “semigood” may include more heterogeneous colonies than other classes. Some of the colonies in class “semigood” may be closer to class “good” than class “bad” and vice versa. Hence, from the classification point of view confusion between pairs semigood-good and semigood-bad may occur more frequently compared to pair good-bad. Confusion between classes again has direct effect on the classification results. By this means in future a possible and a natural continuation would be that classes “bad” and “semigood” would be merged and the classification task would be reduced to binary classification problem. Currently, the reason for the existence of “semigood” class is biological. From the biotechnologists' point of view “semigood” class is reasonable and gives a way to investigate more closely the change of quality in hiPSC colonies. However, as stated earlier from the classification perspective, “semigood” class is difficult and may easily be confused with other classes.

Classification results include great variety and there is not one specific coding design which would have been superior with all classification methods. Every classification method has its specific character and they behave differently with different coding designs. However, the highest accuracy was achieved using ternary complete coding design and *k*-NN classifier. This may indicate that colonies have local similarities between each other and the use of global classification methods might not be the best alternative. Furthermore, ternary complete coding design has the highest number of classifiers and, thus, might have the best separation power since it combines both binary and ternary coding designs. Of course, the downside of ternary complete coding design is the time complexity due to the high number of classifiers.

Finding the best methods for hiPSC colony image classification is an iterative process and here we are concerned only with the classification section. We have found out that *k*-NN classifier seems to be the most appropriate choice for this application. The next step is to return back to the image processing and feature extraction stages and investigate other approaches for this application. Based on [18] and the results it seems that the feature detectors used in computer vision such as SIFT or other closely related techniques are a correct approach for hiPSC colony image classification.

## 6. Conclusions

In this paper, we focused on the automated quality identification of human induced pluripotent stem cell colonies using error-correcting output codes and different machine learning methods. More specifically, we examined four ECOC coding designs and each one of them was tested with four classification methods including their variants. Overall, we performed 120 different test setups and got a wide perspective on how the selected classification approach works with our dataset. As features we used SIFT-based solution which improved the results compared to the earlier studies [16, 17] on this subject where a smaller dataset was used. The best accuracy was 62.4% (the same accuracy was achieved in [5] with *k*-NN without ECOC), being a promising result for the future research. With ECOC we gained improvement on naïve Bayes and classification tree results compared to [5] results. From the application point of view the use of ECOC multiclass modeling is novel since there are no studies made on hiPSC colony classification before where ECOC would have been used. Our paper addresses an important aspect related to hiPSCs. The automation of quality identification of hiPSC colonies is a fundamental issue to be solved in order to enable the use of hiPSCs in practice for different purposes such as disease modeling and tissue repairing. The next step in our research is to investigate bag-of-features [37] approach with different classification methods (e.g., ECOC with SVMs) and textural features extraction methods. In bag-of-features method special attention will be given to codebook size selection and code assignment using different clustering algorithms such as *K*-Means algorithm [23] or DBSCAN [38].

## Figures and Tables

**Table 1:** The coding matrix for one-vs-all (OVA) coding design in the three-class classification problem. In the coding matrix rows represent codewords for each class *C*_*i*_, *i* = 1,2, 3. Columns represent individual classifiers *f*_*i*_, *i* = 1,2, 3, and how classes are divided into positive and negative classes.

	*f*_1_	*f*_2_	*f*_3_
*C*_1_	1	−1	−1
*C*_2_	−1	1	−1
*C*_3_	−1	−1	1

**Table 2:** The coding matrix for the one-vs-one (OVO) coding design in the three-class classification problem.

	*f*_1_	*f*_2_	*f*_3_
*C*_1_	1	1	0
*C*_2_	−1	0	1
*C*_3_	0	−1	−1

**Table 3:** The coding matrix for ordinal (ORD) coding design in the three-class classification problem.

	*f*_1_	*f*_2_
*C*_1_	−1	−1
*C*_2_	1	−1
*C*_3_	1	1

**Table 4:** The coding matrix for ternary complete (TER) coding design in the three-class classification problem.

	*f*_1_	*f*_2_	*f*_3_	*f*_4_	*f*_5_	*f*_6_
*C*_1_	−1	−1	0	1	−1	−1
*C*_2_	1	−1	−1	−1	0	1
*C*_3_	0	1	1	1	1	1

**Table 5:** The results of discriminant analysis variants and classification tree method when different ECOC coding designs have been used. Different coding designs are abbreviated as follows: one-vs-all (OVA), one-vs-one (OVO), ordinal (ORD), and ternary complete (TER). Quadratic discriminant analysis could not be evaluated due to nonpositive definiteness of covariance matrix. True positive rates can be found from the parenthesis next to true positive result and accuracy from the last column of the table.

Method/class	Bad	Good	Semigood	ACC
OVA-LDA	17 (41.5%)	34 (45.9%)	16 (27.6%)	38.7%
OVO-LDA	22 (53.7%)	27 (36.5%)	20 (34.5%)	39.9%
ORD-LDA	17 (41.5%)	24 (32.4%)	18 (31.0%)	34.1%
TER-LDA	16 (39.0%)	32 (43.2%)	20 (34.5%)	39.3%
OVA-diagLinear	19 (46.3%)	**59 (79.7%)**	11 (19.0%)	51.4%
OVO-diagLinear	16 (39.0%)	58 (78.4%)	16 (27.6%)	52.0%
ORD-diagLinear	19 (46.3%)	39 (52.7%)	15 (25.9%)	42.2%
TER-diagLinear	17 (41.5%)	58 (78.4%)	15 (25.9%)	52.0%
OVA-pseudoQuadratic	**41 (100.0%)**	0 (0.0%)	0 (0.0%)	23.7%
OVO-pseudoQuadratic	9 (22.0%)	35 (47.3%)	28 (48.3%)	41.6%
ORD-pseudoQuadratic	41 (100.0%)	0 (0.0%)	0 (0.0%)	23.7%
TER-pseudoQuadratic	9 (22.0%)	31 (41.9%)	**31 (53.4%)**	41.0%
OVA-classification tree	17 (41.5%)	39 (52.7%)	15 (25.9%)	41.0%
OVO-classification tree	19 (46.3%)	50 (67.6%)	30 (51.7%)	**57.2%**
ORD-classification tree	13 (31.7%)	48 (64.9%)	17 (29.3%)	45.1%
TER-classification tree	16 (39.0%)	48 (64.9%)	23 (39.7%)	50.3%

**Table 6:** The results of naïve Bayes variants together with different ECOC coding designs. Different coding designs are abbreviated as follows: one-vs-all (OVA), one-vs-one (OVO), ordinal (ORD), and ternary complete (TER). True positive rates can be found from the parenthesis next to true positive result and accuracy from the last column of the table.

Method/class	Bad	Good	Semigood	ACC
OVA-naïve Bayes (normal distribution assumption)	**17 (41.5%)**	62 (83.8%)	14 (24.1%)	**53.8%**
OVO-naïve Bayes (normal distribution assumption)	16 (39.0%)	61 (82.4%)	14 (24.1%)	52.6%
ORD-naïve Bayes (normal distribution assumption)	16 (39.0%)	49 (66.2%)	**19 (32.8%)**	48.6%
TER-naïve Bayes (normal distribution assumption)	16 (39.0%)	61 (82.4%)	14 (24.1%)	52.6%
OVA-naïve Bayes (kernel smoothing density estimation and triangle kernel)	13 (31.7%)	59 (79.7%)	9 (15.5%)	46.8%
OVO-naïve Bayes (kernel smoothing density estimation and triangle kernel)	13 (31.7%)	59 (79.7%)	12 (20.7%)	48.6%
ORD-naïve Bayes (kernel smoothing density estimation and triangle kernel)	13 (31.7%)	58 (78.4%)	10 (17.2%)	46.8%
TER-naïve Bayes (kernel smoothing density estimation and triangle kernel)	13 (31.7%)	59 (79.7%)	12 (20.7%)	48.6%
OVA-naïve Bayes (kernel smoothing density estimation and Epanechnikov kernel)	15 (36.6%)	60 (81.1%)	10 (17.2%)	49.1%
OVO-naïve Bayes (kernel smoothing density estimation and Epanechnikov kernel)	13 (31.7%)	60 (81.1%)	11 (19.0%)	48.6%
ORD-naïve Bayes (kernel smoothing density estimation and Epanechnikov kernel)	13 (31.7%)	59 (79.7%)	10 (17.2%)	47.4%
TER-naïve Bayes (kernel smoothing density estimation and Epanechnikov kernel)	15 (36.6%)	59 (79.7%)	11 (19.0%)	49.1%
OVA-naïve Bayes (kernel smoothing density estimation and box kernel)	13 (31.7%)	64 (86.5%)	9 (15.5%)	49.7%
OVO-naïve Bayes (kernel smoothing density estimation and box kernel)	12 (29.3%)	**65 (87.8%)**	11 (19.0%)	50.9%
ORD-naïve Bayes (kernel smoothing density estimation and box kernel)	13 (31.7%)	63 (85.1%)	9 (15.5%)	49.1%
TER-naïve Bayes (kernel smoothing density estimation and box kernel)	13 (31.7%)	64 (86.5%)	10 (17.2%)	50.3%
OVA-naïve Bayes (kernel smoothing density estimation and Gaussian kernel)	13 (31.7%)	64 (86.5%)	9 (15.5%)	49.7%
OVO-naïve Bayes (kernel smoothing density estimation and Gaussian kernel)	18 (43.9%)	59 (79.7%)	14 (24.1%)	52.6%
ORD-naïve Bayes (kernel smoothing density estimation and Gaussian kernel)	13 (31.7%)	63 (85.1%)	9 (15.5%)	49.1%
TER-naïve Bayes (kernel smoothing density estimation and Gaussian kernel)	13 (31.7%)	64 (86.5%)	10 (17.2%)	50.3%

**Table 7:** The results of *k*-Nearest Neighbors searching method variants with one-vs-all coding design. True positive rates can be found from the parenthesis next to true positive result and accuracy from the last column of the table.

Method/class	Bad	Good	Semigood	ACC
Chebyshev measure and equal weights	27 (65.9%)	45 (60.8%)	22 (37.9%)	54.3%
Chebyshev measure and inverse weights	15 (36.6%)	44 (59.5%)	21 (36.2%)	46.2%
Chebyshev measure and inverse squared weights	16 (39.0%)	55 (74.3%)	23 (39.7%)	54.3%
Cityblock measure and equal weighting	**28 (68.3%)**	51 (68.9%)	23 (39.7%)	59.0%
Cityblock measure and inverse weighting	25 (61.0%)	52 (70.3%)	**27 (46.6%)**	60.1%
Cityblock measure and squared inverse weighting	24 (58.5%)	50 (67.6%)	**27 (46.6%)**	58.4%
Correlation measure and equal weighting	19 (46.3%)	59 (79.7%)	18 (31.0%)	55.5%
Correlation measure and inverse weighting	16 (39.0%)	54 (73.0%)	19 (32.8%)	51.4%
Correlation measure and squared inverse weighting	19 (46.3%)	58 (78.4%)	21 (36.2%)	56.6%
Cosine measure and equal weighting	24 (58.5%)	52 (70.3%)	19 (32.8%)	54.9%
Cosine measure and inverse weighting	20 (48.8%)	59 (79.7%)	20 (34.5%)	57.2%
Cosine measure and squared inverse weighting	20 (48.8%)	**62 (83.8%)**	21 (36.2%)	59.5%
Euclidean measure and equal weighting	25 (61.0%)	51 (68.9%)	23 (39.7%)	57.2%
Euclidean measure and inverse weighting	24 (58.5%)	49 (66.2%)	25 (43.1%)	56.6%
Euclidean measure and squared inverse weighting	21 (51.2%)	48 (64.9%)	24 (41.4%)	53.8%
Standardized Euclidean measure and equal weighting	**28 (68.3%)**	54 (73.0%)	25 (43.1%)	**61.8%**
Standardized Euclidean measure and inverse weighting	25 (61.0%)	53 (71.6%)	**27 (46.6%)**	60.7%
Standardized Euclidean measure and squared inverse weighting	20 (48.8%)	46 (62.2%)	26 (44.8%)	53.2%
Spearman measure and equal weighting	15 (36.6%)	50 (67.6%)	16 (27.6%)	46.8%
Spearman measure and inverse weighting	16 (39.0%)	61 (82.4%)	17 (29.3%)	54.3%
Spearman measure and squared inverse weighting	18 (43.9%)	59 (79.7%)	19 (32.8%)	55.5%

**Table 8 tab8:** The results of *k*-Nearest Neighbors searching method variants with one-vs-one coding design. True positive rates can be found from the parenthesis and accuracy from the last column of the table.

Method/class	Bad	Good	Semigood	ACC
Chebyshev measure and equal weights	23 (56.1%)	46 (62.2%)	21 (36.2%)	52.0%
Chebyshev measure and inverse weights	23 (56.1%)	46 (62.2%)	21 (36.2%)	52.0%
Chebyshev measure and inverse squared weights	18 (43.9%)	47 (63.5%)	21 (36.2%)	49.7%
Cityblock measure and equal weighting	**24 (58.5%)**	54 (73.0%)	24 (41.4%)	59.0%
Cityblock measure and inverse weighting	**24 (58.5%)**	54 (73.0%)	23 (39.7%)	58.4%
Cityblock measure and squared inverse weighting	21 (51.2%)	52 (70.3%)	20 (34.5%)	53.8%
Correlation measure and equal weighting	17 (41.5%)	54 (73.0%)	18 (31.0%)	51.4%
Correlation measure and inverse weighting	15 (36.6%)	56 (75.7%)	16 (27.6%)	50.3%
Correlation measure and squared inverse weighting	20 (48.8%)	60 (81.1%)	22 (37.9%)	59.0%
Cosine measure and equal weighting	17 (41.5%)	56 (75.7%)	15 (25.9%)	50.9%
Cosine measure and inverse weighting	18 (43.9%)	**64 (86.5%)**	18 (31.0%)	57.8%
Cosine measure and squared inverse weighting	17 (41.5%)	60 (81.1%)	21 (36.2%)	56.6%
Euclidean measure and equal weighting	23 (56.1%)	52 (70.3%)	24 (41.4%)	57.2%
Euclidean measure and inverse weighting	23 (56.1%)	52 (70.3%)	24 (41.4%)	57.2%
Euclidean measure and squared inverse weighting	**24 (58.5%)**	51 (68.9%)	28 (48.3%)	59.5%
Standardized Euclidean measure and equal weighting	23 (56.1%)	58 (78.4%)	23 (39.7%)	60.1%
Standardized Euclidean measure and inverse weighting	23 (56.1%)	58 (78.4%)	23 (39.7%)	60.1%
Standardized Euclidean measure and squared inverse weighting	**24 (58.5%)**	52 (70.3%)	**29 (50.0%)**	**60.7%**
Spearman measure and equal weighting	16 (39.0%)	59 (79.7%)	17 (29.3%)	53.2%
Spearman measure and inverse weighting	18 (43.9%)	61 (82.4%)	19 (32.8%)	56.6%
Spearman measure and squared inverse weighting	18 (43.9%)	62 (83.8%)	21 (36.2%)	58.4%

**Table 9 tab9:** The results of *k*-Nearest Neighbors searching method variants ordinal coding design. True positive rates can be found from the parenthesis next to true positive result and accuracy from the last column of the table.

Method/class	Bad	Good	Semigood	ACC
Chebyshev measure and equal weights	21 (51.2%)	56 (75.7%)	25 (43.1%)	59.0%
Chebyshev measure and inverse weights	21 (51.2%)	56 (75.7%)	25 (43.1%)	59.0%
Chebyshev measure and squared inverse weights	21 (51.2%)	55 (74.3%)	19 (32.8%)	54.9%
Cityblock measure and equal weighting	**23 (56.1%)**	58 (78.4%)	23 (39.7%)	60.1%
Cityblock measure and inverse weighting	**23 (56.1%)**	58 (78.4%)	23 (39.7%)	60.1%
Cityblock measure and squared inverse weighting	23 (56.1%)	56 (75.7%)	24 (41.4%)	59.5%
Correlation measure and equal weighting	16 (39.0%)	45 (60.8%)	15 (25.9%)	43.9%
Correlation measure and inverse weighting	16 (39.0%)	45 (60.8%)	15 (25.9%)	43.9%
Correlation measure and squared inverse weighting	16 (39.0%)	46 (62.2%)	16 (27.6%)	45.1%
Cosine measure and equal weighting	14 (34.1%)	60 (81.1%)	15 (25.9%)	51.4%
Cosine measure and inverse weighting	16 (39.0%)	63 (85.1%)	16 (27.6%)	54.9%
Cosine measure and squared inverse weighting	16 (39.0%)	55 (74.3%)	16 (27.6%)	50.3%
Euclidean measure and equal weighting	16 (39.0%)	55 (74.3%)	19 (32.8%)	52.0%
Euclidean measure and inverse weighting	16 (39.0%)	54 (73.0%)	20 (34.5%)	52.0%
Euclidean measure and squared inverse weighting	18 (43.9%)	54 (73.0%)	18 (31.0%)	52.0%
Standardized Euclidean measure and equal weighting	21 (51.2%)	**64 (86.5%)**	22 (37.9%)	**61.8%**
Standardized Euclidean measure and inverse weighting	21 (51.2%)	63 (85.1%)	22 (37.9%)	61.3%
Standardized Euclidean measure and squared inverse weighting	22 (53.7%)	55 (74.3%)	**26 (44.8%)**	59.5%
Spearman measure and equal weighting	14 (34.1%)	57 (77.0%)	14 (24.1%)	49.1%
Spearman measure and inverse weighting	13 (31.7%)	63 (85.1%)	14 (24.1%)	52.0%
Spearman measure and squared inverse weighting	13 (31.7%)	62 (83.8%)	14 (24.1%)	51.4%

**Table 10:** The results of *k*-Nearest Neighbors searching method variants with ternary complete coding design. True positive rates can be found from the parenthesis next to true positive result and accuracy from the last column of the table.

Method/class	Bad	Good	Semigood	ACC
Chebyshev measure and equal weights	24 (58.5%)	48 (64.9%)	26 (44.8%)	56.6%
Chebyshev measure and inverse weights	22 (53.7%)	46 (62.2%)	24 (41.4%)	53.2%
Chebyshev measure and squared inverse weights	16 (39.0%)	54 (73.0%)	22 (37.9%)	53.2%
Cityblock measure and equal weighting	24 (58.5%)	54 (73.0%)	24 (41.4%)	59.0%
Cityblock measure and inverse weighting	24 (58.5%)	54 (73.0%)	25 (43.1%)	59.5%
Cityblock measure and squared inverse weighting	24 (58.5%)	53 (71.6%)	22 (37.9%)	57.2%
Correlation measure and equal weighting	17 (41.5%)	56 (75.7%)	19 (32.8%)	53.2%
Correlation measure and inverse weighting	17 (41.5%)	59 (79.7%)	22 (37.9%)	56.6%
Correlation measure and squared inverse weighting	19 (46.3%)	57 (77.0%)	22 (37.9%)	56.6%
Cosine measure and equal weighting	17 (41.5%)	59 (79.7%)	17 (29.3%)	53.8%
Cosine measure and inverse weighting	14 (34.1%)	56 (75.7%)	17 (29.3%)	50.3%
Cosine measure and squared inverse weighting	19 (46.3%)	**61 (82.4%)**	21 (36.2%)	58.4%
Euclidean measure and equal weighting	23 (56.1%)	52 (70.3%)	22 (37.9%)	56.1%
Euclidean measure and inverse weighting	24 (58.5%)	51 (68.9%)	**28 (48.3%)**	59.5%
Euclidean measure and squared inverse weighting	22 (53.7%)	50 (67.6%)	25 (43.1%)	56.1%
Standardized Euclidean measure and equal weighting	**25 (61.0%)**	58 (78.4%)	24 (41.4%)	61.8%
Standardized Euclidean measure and inverse weighting	**25 (61.0%)**	58 (78.4%)	25 (43.1%)	**62.4%**
Standardized Euclidean measure and squared inverse weighting	24 (58.5%)	52 (70.3%)	27 (46.6%)	59.5%
Spearman measure and equal weighting	14 (34.1%)	56 (75.7%)	16 (27.6%)	49.7%
Spearman measure and inverse weighting	18 (43.9%)	**61 (82.4%)**	16 (27.6%)	54.9%
Spearman measure and squared inverse weighting	19 (46.3%)	**61 (82.4%)**	20 (34.5%)	57.8%
